# Supplementary material for: TNAP and P2X7R: New Plasma Biomarkers for Alzheimer’s Disease
Source: Int J Mol Sci. 2023 Jun 30;24(13):10897. doi: 10.3390/ijms241310897 (PMC10342008; doi:10.3390/ijms241310897)
Supplement: Supplementary file 1 [file ijms-24-10897-s001.zip › ijms-2419601-supplementary.pdf]

Table S1. Correlation (r) between MRI Fazekas scale and P2X7R levels or %TNAP activity for each diagnostic group.

**MRI Fazekas vs.**	**Control**	**MCI**	**AD**
P2X7R levels	r=-0.143<br>p(two tailed)=0.514 (ns)	r=-0.123<br>p(two tailed)=0.634 (ns)	r=0.156<br>p(two tailed)=0.563 (ns)
%TNAPactivity	r=-0.225<br>p(two tailed)=0.302 (ns)	r=0.167<br>p(two tailed)=0.516 (ns)	r=0.209<br>p(two tailed)=0.434 (ns)

Nonparametric Spearman correlation (r) computed between two selected data sets.

Table S2. Correlation (r) between neuropsychological tests and P2X7R levels or %TNAP activity for control and MCI group.

**MMSE vs.**	**Control**	**MCI**
P2X7R levels	r=-0.095<br>p(two tailed)=0.680 (ns)	**r=0.594**<br>**p(two tailed)=0.028 (*)**
%TNAPactivity	r=0.136<br>p(two tailed)=0.558 (ns)	r=0.132<br>p(two tailed)=0.651 (ns)

**FAQ vs.**	**Control**	**MCI**
P2X7R levels	r=-0.156<br>p(two tailed)=0.500 (ns)	r=-0.395<br>p(two tailed)=0.162 (ns)
%TNAPactivity	r=-0.110<br>p(two tailed)=0.634 (ns)	r=-0.453<br>p(two tailed)=0.105 (ns)

**CDT vs.**	**Control**	**MCI**
P2X7R levels	r=-0.262<br>p(two tailed)=0.251 (ns)	r=0.247<br>p(two tailed)=0.392 (ns)
%TNAPactivity	r=0.093<br>p(two tailed)=0.688 (ns)	r=-0.056<br>p(two tailed)=0.848 (ns)

**GDS vs.**	**Control**	**MCI**
P2X7R levels	r=-0.340<br>p(two tailed)=0.131 (ns)	r=-0.232<br>p(two tailed)=0.421 (ns)
%TNAPactivity	r=0.107<br>p(two tailed)=0.644 (ns)	**r=0.744**<br>**p(two tailed)=0.003(**)**

Nonparametric Spearman correlation (r) computed between two selected data sets.
* $p < 0.05$, ** $p < 0.01$.
